# Vanadium(V) Removal from Aqueous Solutions and Real Wastewaters onto Anion Exchangers and Lewatit AF5

**DOI:** 10.3390/molecules27175432

**Published:** 2022-08-25

**Authors:** Anna Wołowicz, Zbigniew Hubicki

**Affiliations:** Department of Inorganic Chemistry, Faculty of Chemistry, Institute of Chemical Sciences, Maria Curie-Sklodowska University, Maria Curie-Sklodowska Square 2, 20-031 Lublin, Poland

**Keywords:** vanadium, removal, ion exchange resin, wastewater, adsorption

## Abstract

Adsorption abilities of weakly (Purolite A830), weakly basic/chelating (Purolite S984), and strongly basic (Lewatit MonoPlus SR7, Purolite A400TL, Dowex PSR2, Dowex PSR3) ion exchange resins of different functional groups and microporous Lewatit AF5 without functional groups towards vanadium(V) ions were studied in batch and column systems. In the batch system, the influence of the sorbent mass (0.01–0.1 g), pH (2–10), the phase contact time (1–1440 min),and the initial concentration (5–2000 mg/L) were studied, whereas in the column system, the initial concentrations (50, 100, and 200 mg/L) with the same bed volume and flow rate (0.4 mL/min) were studied. Desorption agents HCl and NaOH of 0.1–1 mol/L concentration were used for loaded sorbent regeneration. The pseudo-first order, pseudo-second order and intraparticle diffusion kinetic models as well as the Langmuir, Freundlich, Temkin, and Dubinin–Radushkevich isotherm models were used to describe kinetic and equilibrium data to acquire improved knowledge on the adsorption mechanism. The desorption efficiency was the largest using 0.5 mol/L NaOH for all sorbents under discussion. Purolite S984, Purolite A830, and Purolite A400TL, especially Purolite S984, are characterized by the best removal ability towards vanadium(V) from both model and real wastewater.

## 1. Introduction

**Figure 1:**
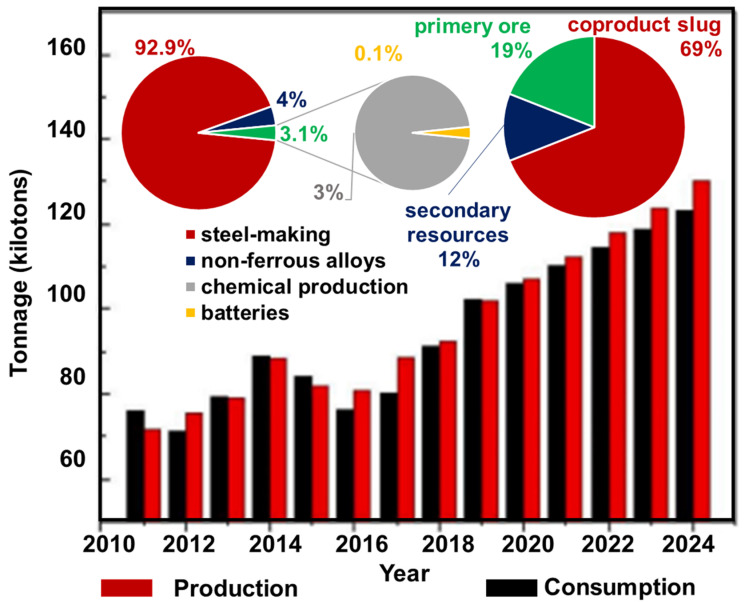
Global vanadium production and consumption in 2010–2025; the sources of vanadium production and branches of industry using vanadium.

Vanadium(V) is a contaminant in wastewater as a result of the economy and industry development as well as increasing consumption year after year (Figure 1). Its enormous application in many fields of industry as well as the mining, smelting, and processing of ores containing vanadium are the main sources of vanadium in the environment (Figure 1) [1].

**Figure 2:**
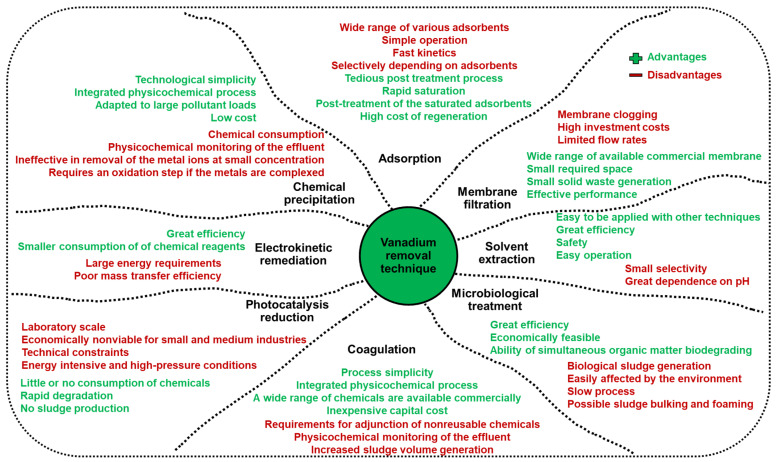
Comparison of treatment technologies for the vanadium removal from wastewaters.

The effluent discharge containing a large concentration of vanadium and its potential toxicity can be dangerous for animals and human [2]. Therefore, increasing attention has focused on the removal of vanadium from water and wastewater to control its concentration in the environment as well as for its recovery. Adsorption, chemical precipitation, solvent extraction, electrokinetic remediation, photocatalysis reduction, coagulation, microbiological treatment, and membrane filtration methods have been developed to remove vanadium from wastewater. The main advantages and disadvantages of the mentioned technique are presented in Figure 2 [3]. In recent years, adsorptions underwent rapid development and became a great potential method for heavy metal removal and separation. Brief consumption, fast kinetics, great effectiveness, technological simplicity, small energy consumption, environmental friendliness as well as the availability of a wide range of adsorbents and possibility of its regeneration render adsorption one of the most frequently applied techniques for vanadium removal [1,2,3,4]. Ion exchange resins, oxides, biosorbents, nanomaterials, and iron-based or hybrid materials were applied for vanadium removal and separation. They are discussed in the literature and take into account solution pH, co-existing ions, contact time, sorbent dosage, temperature, adsorption mechanism, etc. [1,2,3].

Of the commercially available sorbents, ion exchange resins are often used for vanadium removal due to their characteristics of easy operation, small chemical consumption, good flexibility, and versatility [2]. The strongly (Amberlite IRA-400 [5,6], D201, 717 [7], and D296 [8]) and weakly (Amberlite IRA-743 [5], D314 [7,9], ZGA414 [10]) basic anion exchange resins and chelating (Lewatit MonoPlus TP220, Dowex M4195 [11], DDAS, CUW or CW-2 [12]) resins have been applied for vanadium removal in the past [5,6,7,8,9,10,11,12].

The strongly (Amberlite IRA-400) and weakly (Amberlite IRA-743) basic ion-exchange resins were applied for the V(V) and Mo(VI) sorption. The sorption capacity was 208 and 177 mg/L for Amberlite IRA-743 (pH = 4.0) and Amberlite IRA-400 (pH = 6). The sorption process was endothermic [5]. Due to numerous co-properties in the chemistry of molybdenum and vanadium, the separation of these elements is difficult but the D296 resin could be effectively applied for separation in the pH range of 6.5–8.5. The chloride ions affect the separation significantly. When the concentration of the chloride ions is close to 70 g/L, it is impossible to remove vanadium from the solution with D296. The effective regeneration of the loaded strongly basic anion exchange resin over 98.5% was obtained using 6 mol/L HCl. The resin adsorbed V_3_O_9_^3−^ preferentially due to higher charges contained in V_3_O_9_^3−^ than that in MoO_4_^2−^ in the ammonium molybdate solution (pH = 7.14) [8]. Amberlite IRA-400 was also applied for vanadium removal and recovery from the alkaline steel slag leachates at pH 11.5. The sorption kinetics followed the pseudo-first order kinetic model and the maximum adsorption capacity was 27 mg/L. The great anion exchange resin efficiency was also proved in column studies and the desorption yield was 57–72% with 2 mol/L NaOH. The reuse studies showed that this resin could be reused 20 times without a capacity reduction and with 69% of V recovery during the regeneration [6]. Strongly D201, 717, and D296 and weakly ZGA414 and D314 basic anion exchange resins with high efficiency were also confirmed during the vanadium extraction from the hydrochloric acid leaching solution of red mud [7] and from the sulfuric acid leach solutions of stone coals [8,9,10]. More than 99% of vanadium was selectively adsorbed by the D201 resin (pH 2.0, flow speed of 6 m^3^/h·m^3^), D314 (pH 3.0, flow speed of 4.8 m^3^/h·m^3^), and 717 (pH 5.0, flow speed of 7.92 m^3^/h·m^3^) [7] as well as D314 (2.06 g/L (V_2_O_5_), 260 mg/mL, contact time of 60 min, pH 4) [9]. The adsorption process of vanadium follows the pseudo-second order kinetic model [7]. The separation of vanadium from the solution containing Fe(III), Fe(II), Al(III), and Mg(II) ions by the ion exchange and the solvent extraction indicated that only V(V) was loaded from the synthetic solution by ZGA414 at pH > 1.5, and after the reduction to V(IV), vanadium was extracted with 99% efficiency [10].

Vanadium and chromium separation from the vanadium titanomagnetite was also performed using the macroporous weakly basic D314 anion exchange resin [13]. The separation was completed in the pH range of 2.5–6.5 by double adsorption. On the other hand, double adsorption could increase the reagent cost and the operation time results limiting its practical application. To minimize the total cost of vanadium and chromium separation, the pretreatment method was applied [14]. V(V) and Cr(VI) were reduced with NaHSO_3_ (pH 1.7); then, VO^2+^ ions were oxidized by H_2_O_2_ to the H_2_V_10_O_28_^4−^ anions (pH 3.5); on the other hand, Cr^3+^ ions were not oxidized, and after that, V and Cr were separated by the Dex-V anion exchange resin due to different charge natures.

Based on many examples of vanadium removal and separation by the ion exchange resins of excellent physical and chemical stability (strongly basic anion exchange resins) and regenerative and reuse capabilities (weakly basic anion exchange resins), their adsorption capabilities are strongly affected by pH and co-existing inorganic ions [1]. Therefore, the choice of proper ion exchange resins for vanadium removal from wastewater is precisely dependent on wastewater composition, co-ions and its concentration, and should be individually selected.

The aim of the studies was to determine the efficiency of V(V) ion removal from the model’s aqueous solutions and real wastewater (obtained after the digestion of spent catalyst for sulfuric acid production) using ion exchangers of weakly (Purolite A830, abbr. A830; abbr.—abbreviation), weakly basic/chelating (Purolite S984, abbr. S984), and strongly basic (Lewatit MonoPlus SR7, abbr. SR7; Purolite A400TL, abbr. A400TL; Dowex PSR2, abbr. PSR2; Dowex PSR3, abbr. PSR3) ion exchange resins of different functional groups and microporous Lewatit AF5 (abbr. AF5) without functional groups for comparison. These ion exchangers and the AF5 sorbent adsorption ability toward vanadium have not been described in the literature yet, and the studies fill in the gap developing knowledge about vanadium removal. The optimization of the adsorption process: the adsorbent mass, phase contact time, pH, initial concentration of V(V) ions, and the determination of the maximum adsorption capacities as well as the desorption studies were presented. In this paper, kinetic and equilibrium models were used to describe the vanadium adsorption process and a possible adsorption mechanism was proposed.

## 2. Results

### 2.1. Sorbent Characteristics

The sorbents selected for vanadium removal are macroporous or microporous, including polystyrene, polyacrylic matrices with different functional groups (FG) or a carbonaceous matrix without FG. The physicochemical properties were presented based on the producer data sheets of SR7, A400TL, PSR2, PSR3, A830, S984, and AF5 sorbents as well as from our previous studies [15,16], and they are collected in Table 1.

**Figure 3:**
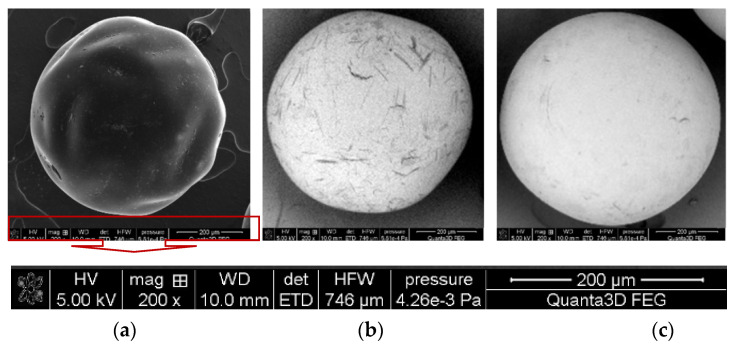
Chosen examples of SEM images of (**a**) AF5, (**b**) A830, and (**c**) SR7 (magn. 200×).

**Figure 4:**
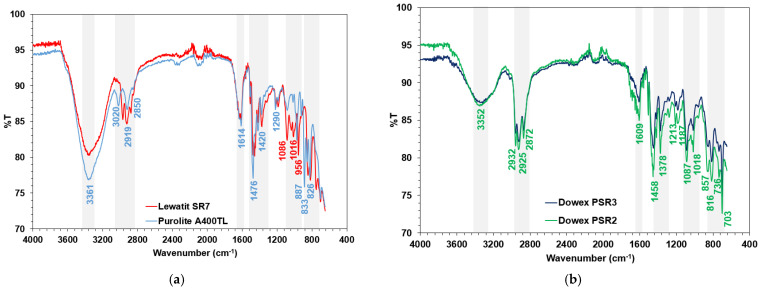

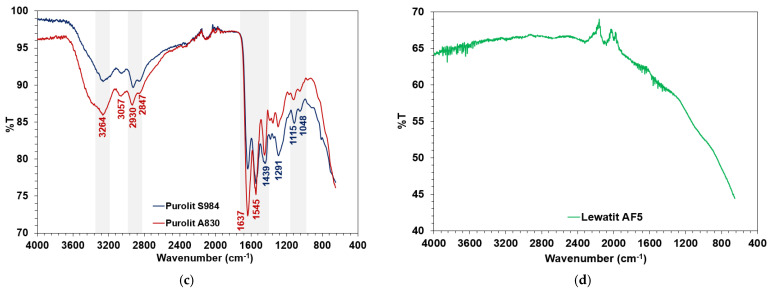
ATR-FTIR spectra: (**a**,**b**) SBA resins, (**c**) WBA resins, and (**d**) AF5 applied for vanadium (V) removal.

The chosen examples of SEM images of the sorbents under discussion are presented in Figure 3. All ion exchangers are in the form of round beads of proper size while AF5 is also in the form of beads that are not ideally spherical. This observation and our previous studies also proved the porosity of the ion exchangers. The presence of the functional groups and matrices of the sorbent was proved by using Attenuated Total Reflectance Fourier Transform Infrared Spectroscopy (ATR-FTIR) studies (Figure 4).

In the ATR-FTIR spectra of ion exchangers before the vanadium(V) sorption process (Figure 4a–c), broad bands of large intensity corresponding to the stretching vibrations of the –OH and =N–H groups were observed at about 3100–3700 cm^−1^ [16]. For all anion exchange resins, this peak is observed but the minimum of peak is at wavenumbers 3361 cm^−1^ for A400TL, 3351 cm^−1^ for SR7, 3352 cm^−1^ for PSR2, 3344 cm^−1^ for PSR3, 3264 cm^−1^ for A830, and 3260 cm^−1^ for S984. This wide band indicates the presence of water in the ion exchanger phase [17]. The peaks originating from the ion exchangers matrices (cross-lined polystyrene or cross-linked polyacrylic) were also found in Figure 4a–c. In the wavenumber range of 3000 and 3100 cm^−1^ as well as 2800 and 3000 cm^−1^, there were observed narrow bands of high intensity resulting from the aromatic vibrations of the C-H groups as well as the aliphatic symmetric (ν_s_) and asymmetric (ν_as_) C–H stretching vibrations of the –CH_2_ group [17,18,19]. These peaks (Figure 4a,b) are located at 3020 cm^−1^, 2919 cm^−1^, and 2850 cm^−1^ for A400TL; at 2936 cm^−1^, 2923 cm^−1^, and 2876 cm^−1^ for SR7; and at 2932 cm^−1^, 2925 cm^−1^, and 2872 cm^−1^ for PSR2 and PSR3. In the case of WBA resins (Figure 4c), these peaks are less sharp and are located at 3057 cm^−1^, 2930 cm^−1^, and 2847 cm^−1^ for A830 and at 3046 cm^−1^, 2923 cm^−1^, and 2867 cm^−1^ for S984. Bands in the wavenumber ranging from 1650 to 1400 cm^−1^ were also observed (Figure 4a–c). These bands correspond to carbon–carbon stretching skeletal vibrations inside the benzene ring and C-H groups connected to the aromatic ring [17]. In the wavenumber range of 1378–1458 cm^−1^, bands related to the deformation vibrations in the –CH_2_ group, which are characteristic of the polymer matrix of the ion exchangers, were observed [17,19]. For the SBA resins (A400TL, SR7, PSR3, and PSR2), the characteristic bands located at 830–890 cm^−1^, e.g., 887, 833, and 826 cm^−1^ for A400TL originate from quaternary ammonium functional groups, R–N^+^–(CH_3_)_3_, stretching vibrations, and the C-Cl stretching vibration of the CH_2_Cl groups, whereas for WBA resins, these bands were not observed [18]. Additionally, for SBA resins, the peaks at 1460–1480 cm^−1^ of the C–N vibrations of R–N^+^–(CH_3_)_3_ appeared [18,20]. In Figure 4d the spectra of Lewatit AF5 are present by the ATR-FTIR method. In these spectra, the peaks for SBA and WBA resins were not observed. There are no of peaks that come from the functional groups of the sorbent, and the spectra are different compared to that presented in Figure 4a–c. This sorbent belongs to different types thananion exchange resins and exists in the form of black beads. Moreover, the shape of spectra could be a result of some restriction of ATR-FTIR for samples of high index of refraction (close to that of diamond). The spectrum depends upon the depth of penetration (d_p_), which in turn depends on other factors, e.g., the ratio of the index of refractions of the sample and crystal. If the sample index is too close to that of the crystal, the ratio of indices is almost 1, the square root term is negative, and there is no ATR effect [21]. The carbonaceous matrix of the Lewatit AF5 was proved by the XRD studies presented in our previous paper [22] and by the %CNHS analysis.

### 2.2. Adsorption Studies–Static and Dynamic Methods

The adsorption studies of vanadium(V) removal were carried out using static and dynamic methods. The adsorption efficiency of SR7, A400TL, PSR2, PSR3, A830, S984, and AF5 sorbents towards V(V) was estimated with the percentage removal (*%R*) (Equation (1)), the sorption capacity (*q_e_*) (Equation (2)), the amount of V(V) adsorbed after time *t* (*q_t_*) (Equation (3)) values (the static studies), and the weight (*D_w_*) (Equation (4)) and bed (*D_b_*) (Equation (5)) distribution coefficients as well as the working ion exchange capacity (*C_w_*) (Equation (6)) (the column studies) (Table 2).

#### 2.2.1. Batch Adsorption—Effect of Adsorbent Doses

**Figure 5:**
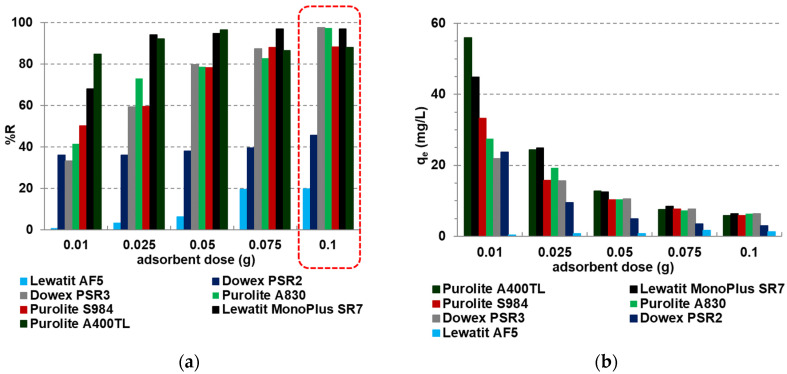
Effects of sorbent mass on (**a**) the percentage removal (*%R*), (**b**) sorption capacity (*q_e_*) values obtained during the V(V) sorption on the sorbents under discussion.

The dose of the sorbent is an important parameter affecting the number of sorption sites of the sorbents, their surface areas, and economical costs of adsorption [2]. Studies on the effects of the adsorbent dose (0.01 to 0.1 ± 0.0005 g) on the vanadium(V) adsorption process were carried out at the initial concentration of V(V) ions *C*_0_ = 50 mg/L, temperature T of 295 K with the agitation speed 170 rpm, the amplitude, *A* = 8, and the phase contact time, *t* = 4h, according to the batch method described in Section 3.2. The adsorption efficiency results are presented in Figure 5. The plot of the sorbent dose versus the percentage removal (Figure 5a) shows that the *%R* values increased from 67.9 to 97% for SR7, from 84.8 to 88% for A400TL, from 41.6 to 97.4% for A830, from 36 to 45.5% for PSR2, from 33.2 to 97.7% for PSR3, from 50.3 to 88.2% for S984, and from 0.5 to 19.8% for AF5 with the increase in the sorbent mass from 0.01 to 0.1 g. On the other hand, adsorption capacities decrease with sorbents’ mass increase, e.g., from 44.9 to 6.4 mg/L for SR7, from 56 to 5.82 mg/L for A400TL, from 27.5 to 6.4 mg/L for A830, from 23.8 to 3.0 mg/L for PSR2, from 22.0 to 6.4 mg/L for PSR3, and from 33.2 to 5.8 mg/L for S984. In the case of Lewatit AF5, *q_e_* values slightly increased from 0.4 to 1.7 mg/L and then decreased to 1.3 mg/L. With the sorbent’s mass increase, the surface area and number of the active sites increase. At the same initial concentration of V(V) during the sorption, the number of unsaturated adsorption sites increases with increasing sorbent mass. Taking into account the values of *%R*, the optimal sorbent mass selected for further research is 0.1 g.

Generally, on the one hand, the adsorption efficiency increases with the increase in adsorbent dosage; the number of active sites and its availability are greater. However, on the other hand, the adsorption capacity per unit weight of the sorbent decreases with the increase in sorbent doses—the interactions of active sites of the sorbent with the removal contaminants [2,11,17]. For example, the V(V) removal efficiency reached 97.79% for Amberlite IRA743, 95.21% for Lewatit MK51, 84.72% for Purolite S110, and 99.89% for Purolite S108 by increasing the dosage of macroporous ion exchangers (the polystyrene matrix cross-linked with divinylbenzene with the N-methyl-D-glucamine groups) from 0.05 to 0.20 g, but the sorption capacities decreased from 18.70 to 11.36 mg/g for Amberlite IRA743, from 24.53 to 11.06 mg/g for Lewatit MK51, from 19.23 to 9.85 mg/g for Purolite S110, and from 34.86 to 11.61 mg/g for Purolite S108 with the increase in the adsorbent’s mass from 0.05 to 0.20 g. In this case, the optimal adsorbent mass was 0.05 g [17]. Similar observations were also made in our previous studies of V(V) removal by Lewatit MonoPlus TP220 and Dowex M4195 of bis-picolylamine functional groups. In that case, the optimal adsorbents mass was also 0.1 g [11]. The increase in the biochar-stabilized nano-zero-valent iron (nZVI/BC) in dose from 0.2 to 1.5 g/L shows that the V(V) removal performance was not satisfactory at doses smaller than 0.5 g/L (removal efficiency 79%), and it increased to 98% with the dose of nZVI/BC, increased to 0.5 g/L, and reached 100% at the dosage above 0.8 g/L. At the same time, the adsorption capacity of V(V) decreased from 48.2 mg/g to 31.4 mg/g. The optimal sorbent dose was selected to be 0.5 g/L [23]. The literature reports that in some cases, the vanadium removal efficiency despite the increasing sorbent dose is unchanged [24] or the increasing adsorbent dose changes the solution’s pH; e.g., pH decreases with an increasing dosage of ferric oxyhydroxide, CFH-12 (leaching of impurities), or increases with an increasing dosage of raw zeolite (leaching of hydroxides from the pores due to the alkaline-based synthesis procedure) [24,25].

#### 2.2.2. Batch Adsorption—Effects of Solutions pH

**Figure 6:**
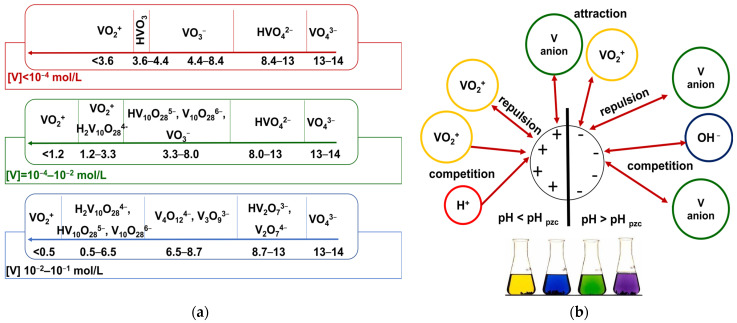
Vanadium species (**a**) and possible interactions with an adsorbent (**b**) depending on its concentration (**a**), pH (**a**,**b**), and surface charge (**b**).

Solution pH can influence directly on both the adsorbent properties (surface charge) and vanadium speciation in the aqueous solutions and simultaneously on the vanadium removal efficiency [26]. The surfaces of the adsorbents could be negatively or positively charged when pH > pH_pzc_ (repulsion between the vanadium anion and the sorbent surface) or pH < pH_pzc_ (positively charged surface favor adsorption of the vanadium anion). In the aqueous solutions vanadium(V) exists in the form of different soluble species depending on pH and log of the total vanadium concentrations [27,28,29,30]. Cationic, neutral and anionic species as well as the other mono- or poly-vanadate ones can be found (Figure 2 in [11], Figure 6a). At low pH (pH < 3) vanadium exists mostly as the cationic species (VO_2_^+^) whereas at higher pH values (pH >3) there exist the anionic species such as the monovanadate ones (H_2_VO_4_^−^, HVO_4_^2−^, VO_4_^3−^ as the site of protonation is oxygen therefore more precise representation would be VO_2_(OH)_2_^−^, VO_3_(OH)^2−^, VO_4_^3−^), the polyvanadate species HV_2_O_7_^3−^, V_2_O_7_^4−^, V_3_O_9_^3−^, V_4_O_12_^4−^) and the decavanadate species (H_2_V_10_O_28_^4−^, HV_10_O_28_^5−^, V_10_O_28_^6−^) [27,28,31]. At the low vanadium concentration the monomeric species are observed whereas with the increasing V concentration condensation process occurs [31].

**Figure 7:**
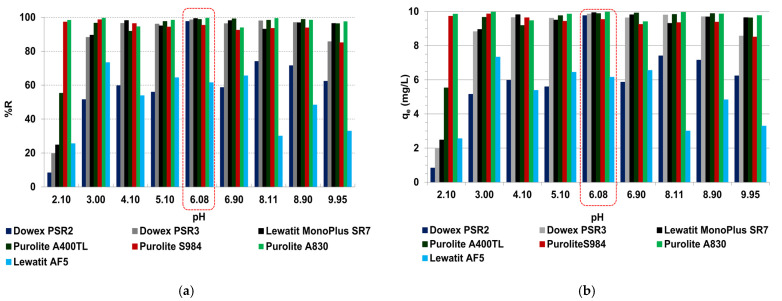
Effects of solutions pH on (**a**) the percentage removal (*%R*), (**b**) sorption capacity (*q_e_*) values obtained during the V(V) sorption on the sorbents under discussions (50 mg V(V)/L, 4 h, 0,1 g).

The effects of solutions pH (2–10) on the vanadium(V) removal efficiency on the SR7, A400TL, PSR2, PSR3, A830, S984, and AF5 sorbents are presented in Figure 7 (0.1 g, *C*_0_ = 50 mg/L, T = 295 K, 170 rpm, *A* = 8, *t* = 4 h). As observed, the smallest adsorption capacities were obtained for pH < 3 as a result of (a) the electrostatic repulsion of main dominant VO_2_^+^ species in the solutions of pH < 3 between vanadium and the positive charges of the sorbent surface and (b) the competition of H^+^ ions with VO_2_^+^ for the active site on the adsorbent results in adsorption efficiency reductions [7] (Figure 6b). As it was pointed out by Bello et al., the small sorption capacity of zero-valent iron (ZVI) conjugating with the kaolin clay (Slu-KZVI) sorbent towards vanadium in the solution of pH < 3 could be also a result of the destruction of the adsorbent structure at a very small pH and the release of adsorbed vanadium into the solution [32].

Most SR7, A400TL, PSR2, PSR3, A830, S984, and AF5 sorbents exhibit the great sorption capacities at pH 3–9, and the highest values were observed at pH ≈ 6; therefore, this pH was chosen in further studies. Moreover, for the WBA resins A830 and S984 (WBA/chelating), the capacities are high in the entire range of examined pH 2–10. In acidic solutions, the functional groups of WBA resin could be protonated [33]. At pH 3–9, vanadium exists in the form of polynuclear anions, e.g., V_2_O_6_(OH)^3−^, V_2_O_7_^4−^, V_3_O_9_^3−^, and V_4_O_12_^4−^; therefore, the interaction of the vanadium anionic species and the anion exchange resins occurs [2]. The possible mechanism of vanadium adsorption could be through the ligand and anion exchange with the functional groups on the adsorbents [9]. The ion exchange reactions of vanadium adsorption on the resin in the Cl^-^ form can be expressed as follows.

R_3_N + HCl = (R_3_NH)Cl (7)

4(R_3_NH)Cl + H_2_V_10_O_28_^4−^ = (R_3_NH)_4_H_2_V_10_O_28_ + 4Cl^−^ (8)

5(R_3_NH)Cl + HV_10_O_28_^5−^ = (R_3_NH)_5_HV_10_O_28_ + 5Cl^−^ (9)

6(R_3_NH)Cl + V_10_O_28_^6−^ = (R_3_NH)_6_V_10_O_28_ + 6Cl^−^ (10)

The electrostatic attractions between the positively charged surface groups of the adsorbent and the anionic vanadium species favour V adsorption [34]. At pH 9, the adsorption capacities decrease as a result of the competition effect between the OH^-^ ions and V anions of the adsorption sites (Figure 7) [35]. Additionally, at high pH, vanadium polynuclear species are converted to mononuclear ones; therefore, more adsorption sites are needed for V adsorptions [34]. Similarly to our findings, the literature reports that the maximum adsorption capacities for vanadium were acquired at pH 3–9 [2]. The optimal pH values selected for vanadium adsorptions were pH 11.5 for SBA Amerlite IRA400 (*%R* = 99.6%) [36], pH 7 for the cation exchanger Amberjet™ 1200 H (*%R* = 70–75%) [24], pH 7 for anion exchanger Amberjet™ 4200 Cl (*%R* = 89%) [24], pH 4–7 for the Amberlite IRA-904 modified with tetrakis (p-carboxyphenyl) porphyrin (*%R* > 80%) [37], etc.

#### 2.2.3. Batch Adsorption—Effects of Contact Time

**Figure 8:**
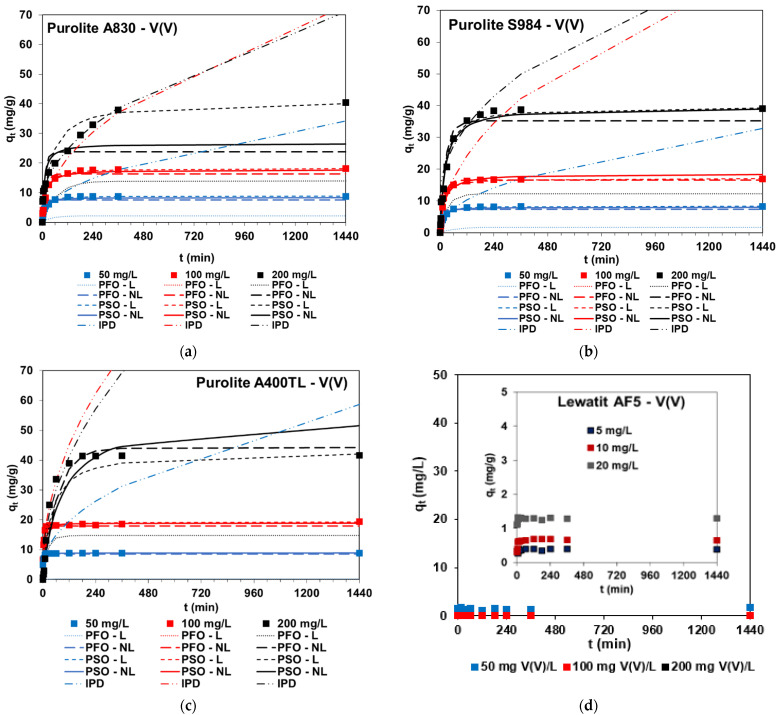
Effects of phase contact time and initial concentration on the amount of vanadium adsorbed on (**a**) A830, (**b**) S984, (**c**) A400TL, and (**d**) AF5.

The phase contact time is an important factor affecting both adsorption performance and adsorption economy; therefore, the vanadium adsorption effectiveness was analyzed by also taking into account these factors. The phase contact time was in the range from 1 min to 24 h (0.1 ± 0.0005 g, *C*_0_ = 50, 100, 200 mg V(V)/L for SR7, A400TL, PSR2, PSR3, A830, S984 or 5, 10, 20, 50, 100 mg/V(V)/L for AF5, *V* = 20 mL, pH ≈ 6, 170 rpm, *A* = 8, *T* = 295 K). The kinetic curves for different initial concentrations of V(V) ions are presented in Figure 8a–d (chosen examples). In the case of the Lewatit AF5 sorbent without functional groups at an initial vanadium concentration of 100 mg/L, vanadium removal was not observed (Figure 8d); therefore, much smaller initial concentrations at 5, 10, and 20 mg/L were selected. Compared with the other sorbents in the selectivity series, this sorbent takes the last position and shows the smallest adsorption capacities at 0.4 mg/g for *C*_0_ = 5 mg/L, 0.7 mg/g for *C*_0_ = 10 mg/L, 1.3 mg/g for *C*_0_ = 20 mg/L, and 1.6 mg/g for *C*_0_ = 50 mg/L (Figure 8d).

Based on the kinetic studies, the following can be concluded:

➢ Vanadium kinetic plots usually included two stages: (1) fast adsorption due to great accessibility of free adsorption sites and (2) slower adsorption stage and reaching equilibrium—the remaining adsorption site’s availability is more difficult [17]. In the case of 200 mg V(V)/L concentrations, three stages can usually be distinguished.
➢ The amount of V(V) ions adsorbed increase with the increasing phase contact time, e.g., *q_t_* = 5 mg/g (*t* = 1 min) and *q_t_* = 8.7 mg/g (*t* = 30 min) for *C*_0_ = 50 mg/L and A400TL, but the shape of the kinetic curves and their “steepness” depend on the initial concentrations of V(V) ions present in the solution.
➢ The adsorption capacities usually increase with increasing initial concentrations, e.g., *q_e_* = 8.8 mg/g (*C*_0_ = 50 mg/L), *q_e_* = 19.3 mg/g (*C*_0_ = 100 mg/L), and *q_e_* = 40.1 mg/g (*C*_0_ = 20 mg/L) for A400TL.
➢ The time required for equilibrium establishment depends on the initial vanadium concentration: longer times are needed for the equilibrium establishment for systems containing 100 and 200 mg V(V)/L compared to 50 mg V(V)/L. Comparing the equilibrium time for vanadium removal (*t_eq_*) by various sorbents, e.g., SBA resin Amberlite IRA-400 (*t_eq_* = 5 min) [6], SBA resin Amberlite IRA-400 (*t_eq_* = 60 min) [5], SBA resin D296 (*t_eq_* = 12 h) [8], WBA resin 201*7 (*t_eq_* = 20 min) [38], WBA resin ZGA414 (*t_eq_* = 8 min) [10], nano-hydrous zirconium oxide-modified anion exchange resin, (*t_eq_* = 2 h) [34], N235 impregnated resin (NIRs) (*t_eq_*=10 h), N235-TBS-impregnated resin (N-TIRs) (*t_eq_* = 6 h) [39], di-(2-ethylhexyl) phosphoric acid (D2EHPA)-Tributyl phosphate (TBP)-impregnated resin (*t_eq_* = 36 h) [40] and that presented in the review paper in Table 2 [2] and taking into account this paper, *t_eq_* is highly variable, ranging from a few minutes to 48 h.
➢ With the higher V(V) concentration, the percentage removal of vanadium is smaller, e.g., 99.9%, 98.6%, 94.4% for 50, 100, and 200 mg V(V)/L, and A400TL or 38.9%, 33.0%, 32.7%, and 18.5% for 5, 10, 20, and 50 mg V(V)/L, and AF5, respectively.

From the practical point of view and considering the applicability of sorbents in industry, the obtained experimental kinetic data were modeled with commonly used kinetic equations such as the pseudo-first order kinetic equation (PFO), the pseudo-second order kinetic equation (PSO) as well as the intraparticle diffusion (IPD) to describe the V(V) adsorption process:

PFO: non-linear (Equation (11)), linear (Equation (12)):

(11) $$q_{t}=q_{e}\left(1-e^{-k_{1}t}\right)$$

(12) $$\log\left(q_{e}-q_{t}\right)=\log q_{e}-\frac{k_{1}}{2.303}t$$

where *k*_1_ = −2.303 × slope, *q_e_* = 10^intercept^, *q_e_* and *q_t_* (mg/g) are the amounts of V(V) sorbed at the equilibrium and at any time *t*, *k*_1_ (1/min) includes the rate constants determined from the PFO equation, and *t* denotes the contact time [41]:

PSO: non-linear (Equation (13)), linear (Equation (14)):

(13) $$q_{t}=\frac{q_{e}^{2}k_{2}t}{q_{e}k_{2}t+1}$$

(14) $$\frac{t}{q_{t}}=\frac{1}{k_{2}q_{e}^{2}}+\frac{1}{q_{e}}t$$

where *k*_2_ (g/mg min) denotes the rate constants determined from the PSO equation, *k*_2_ = slope^2^/intercept, *q_e_* = 1/slope, *h* denotes the initial sorption rate, and $h=k_{2}q_{e}^{2}$ [42]:

IPD: linear (Equation (15)):

(15) $$q_{t}=k_{i}t^{1/2}+C$$

where *k_i_* (mg/g min^0.5^) denotes the intraparticle diffusion rate constant, *k_i_* = slope, *C* denotes the Weber–Morris diffusion constant, and *C* = intercept [43].

The obtained kinetic parameters using the PFO, PSO, and IPD models with linear (L) and non-linear (NL) regressions are presented in Table 3. Due to the kinetic curve’s shape and experimental points position (AF5) and smaller than 10 mg/g sorption capacity for 50 and 100 mg V(V)/L (SR7, PSR2, and PSR3), the kinetic parameters were not calculated (AF5) or calculated but not presented in Table 3 (50, 100 mg/L for SR7, PSR2, PSR3).

**Figure 9:**
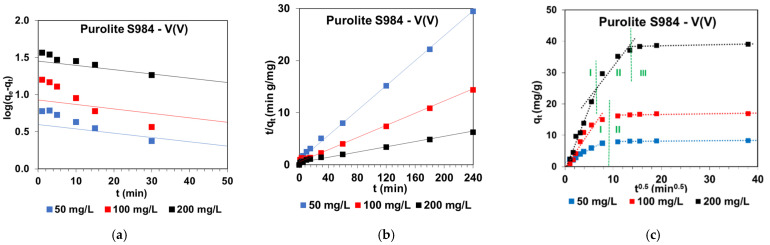
Examples of (**a**) PFO, (**b**) PSO, and (**c**) IPD plots obtained during the V(V) adsorption on Purolite S984.

The Lagergren equation did not find applicability for the description of the V(V) adsorption kinetics on the sorbents under discussion due to the small values of the determination coefficients *R*^2^ being in the range of 0.439–0.985 (L) and ≤ 0.991 (NL). In addition, the calculated equilibrium capacities were much smaller than those determined experimentally (Figure 8). Moreover, the graph *log(q_e_ − q_t_)* vs. *t* was not linear (e.g., Figure 9a).

Figure 9b shows the example of the linearized form of the PSO kinetic dependence for V(V) adsorption from the solutions of different initial concentrations on Purolite S984 and the plot *t/q_t_* vs. *t* shows the linear relationship. Moreover, based on the data presented in Table 3 and Figure 8, it can be concluded that due to large values of the determination coefficient for PSO-NL or very large values for PSO-L and great agreement between the adsorption capacity determined experimentally and calculated from PSO-L, the PSO-L model received the best fit to the experimental data for SR7, A400TL, PSR2, PSR3, A830, and S984 sorbents.

The IPD plots obtained for the SR7, A400TL, PSR2, PSR3, A830, and S984 sorbents show the applicability of the intraparticle diffusion model in the V(V) adsorption if the plot of *q_t_* vs. *t*^1/2^ provide a straight line and passes through the origin. If these factors are fulfilled, the intraparticle diffusion is the only rate-controlling step of the vanadium adsorption process; otherwise, it is not the only rate limiting step. The obtained IPD plots (e.g., Figure 9c) illustrate multi-linearity. The adsorption data can be fitted with two (A830) or three (SR7, A400TL, PSR2, PSR3, and S984) straight lines. The proper parts of the IPD graph correspond to the diffusion effects by the boundary layer or intraparticle diffusion (diffusion into the polymer network). As it was found, the IPD plots do not pass through the origin in all cases, indicating that apart from the intraparticle diffusion, other factors also have an influence on the adsorption rate. Similar observations were also made by other research studies during the V(V) adsorption on various sorbents, e.g., Burdzy et al. [17], Stanisz et al. [44], and Kajjumba et al. [45] (please see also column kinetic studies in Table 4).

### 2.3. Equilibrium Studies

The experimental data for vanadium adsorption on the S984, A830, A400TL, SR7, PSR2, PSR3, and AF5 sorbents were fitted with the Langmuir, Freundlich, Temkin, and Dubinin–Raduskievich adsorption isotherms (description of isotherms are included in Table 5). The Langmuir isotherm described sorption on the homogenous surface with a finite, limited number of adsorption sites and constant energy without interactions between the sorbed molecules [47]. The Freundlich adsorption isotherm describes the multilayer adsorption on the heterogeneous adsorbent surface (non-ideal adsorption) [36,48]. The sorption centers with the highest sorption energy will be saturated first, and then the centers with increasingly smaller energies are saturated. Due to possible interactions between the adsorbed molecules, the variation of adsorption heat may proceed [49]. The Temkin isotherm model describes the adsorption on a heterogeneous solid and takes into account adsorbate–adsorbent interactions. This model assumes that the heat of adsorption of all molecules in the layer decreases linearly, and the adsorption is characterized by the uniform distribution of binding energy [49,50]. The Dubinin–Radushkevich isotherm model is often used to estimate the free energy of adsorption, which could indicate the physical or chemical adsorption mechanism depending on its values. When *E* is in the range from 1 to 8 kJ/mol, the adsorption is a result of physical interactions, while an E between 8 and 16 kJ/mol indicates the ion exchange, and E >16 kJ/mol suggests chemical adsorptions [49,51].

**Figure 10:**
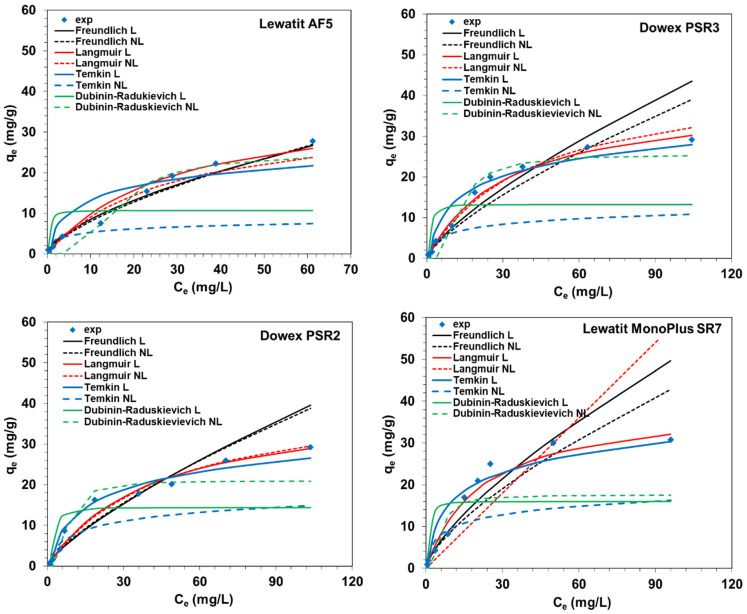
Examples of the isotherms fitting to the experimental data for the sorption of V(V) ions on SR7, PSR2, PSR3, and AF5 sorbents using L (linear) and NL (non-linear) regressions.

Characteristic parameters of the obtained Langmuir, Freundlich, Temkin, and Dubinin–Raduskievich isotherm models by linear regression are presented in Table 5 (obtained by the linear regression), whereas the selected examples of fitting plots (linear—L; non-linear—NL regression) are depicted in Figure 10.

As follows from Table 5, the comparison of correlation coefficient *R*^2^ values obtained for the models shows that the Langmuir model fits well with the experimental data for S984, A830, and A400TL (*R*^2^ values range from 0.927 to 1.000). In the case of SR7, both Langmuir and Freundlich models produce similar values of *R*^2^ at 0.959 and 0.963, respectively, whereas for PSR2 and PSR3, the *R*^2^ values obtained based on the three isotherm Langmuir, Freundlich, and Temkin models are similar in the range from 0.940 to 0.954 for PSR2 and from 0.924 to 0.962 for PSR3. The Dubinin–Raduskievich isotherm model generally did not find applicability for the description of the V(V) adsorption on all sorbents under discussion. For AF5, the Freundlich isotherm model is the best fitting. Moreover, when the correlation coefficients for the Langmuir model are high, a high agreement between the adsorption capacity values obtained experimentally and those determined from the model can also be observed. Comparing the values of the adsorption capacities calculated based on the proper isotherm models included in Table 4 [4,11,17,36,46] and the other ion exchangers (e.g., the weakly basic anion exchange resins: 717 (*q_max_* = 18.54 mg/g), D314 (*q_max_* = 18.08 mg/g), D201 (*q_max_* = 18.02 mg/g), and 201*7 (*q_max_* = 48.0 mg/g) [2] as well as the carbon adsorbents, e.g., sawdust biochar (*q_max_* = 1.5 mg/g), commercial activated carbon (AC) (*q_max_* = 7.58 mg/g), ZnCl_2_-corn straw biochar (*q_max_* = 26.26 mg/g), CsCl-corn straw biochar (*q_max_* = 43.28 mg/g), Zr(SO_4_)_2_-corn straw biochar (*q_max_* = 58.74 mg/g), ZnCl_2_-coir pith derived (*q_max_* = 24.9 mg/g), Norit ROY 0.8 AC (*q_max_* = 37.87 mg/g), etc.) [4], it can be concluded that S984, A830, and A400TL are very promising in vanadium removal due to the exhibited largest adsorption capacities.

The adsorption mechanism of vanadium on different types of sorbents is different and depends strictly on the adsorbate and adsorbent type as well as the pH and experimental conditions of the adsorption process. The electrostatic interactions, ligand-exchange, ion-exchange, chelation, redox mechanism, or mixed interactions can be distinguished.

Electrostatic interactions proceed between anionic species of vanadium and the positively charged adsorbents. Depending on the solution’s pH, the surface charge on the adsorbents can be changed as a result of the protonation and deprotonation of some surface functional groups. With the solution’s pH increase, adsorbent surfaces tend to be more positively charged and stronger electrostatic attraction for the V anions can occur; e.g., the increased H^+^ ions concentration resulted in the protonation of the surface hydroxyl groups (-OH_2_^+^) of the double-layered hydroxide-supported nanoscale zero-valent iron, leading to electrostatic attraction improvements [26]. On the other hand, electrostatic interactions could proceed between the cations, e.g., Na^+^, Mg^2+^, Ca^2+^, etc., adsorbed on the adsorbent surface, which form a bridge of a positive charge on the surface with the anionic vanadium species present in the solutions [52]. During the vanadium adsorption on the Ti-doped chitosan bead, the ligand exchange, electrostatic interaction, and redox reaction are the three main mechanisms responsible for vanadium adsorption (Ti^4+^ with the positive charge formed by the protonation results in stronger attraction towards the vanadium anions H_3_V_2_O_7_^−^ or H_2_VO_4_^−^). The Cl^-^ ligand on the TiCB surface can also exchange with these anionic species and form the Ti–H_3_V_2_O_7_ or Ti–H_2_VO_4_ complex; then, the surface of TiCB might be partially reduced, and the free V(IV) might be re-adsorbed to form the TiCB–V(IV) complex [53]. Kończyk et al. [4] suggested that the V(V) anions adsorbed onto BC2 biochar by the anion exchange, electrostatic interactions, H-bond formation mechanism, etc., and the functional groups of biochar participated in V adsorption. Moreover, the inner-sphere complexes’ formation may occur during vanadium adsorption on iron-based materials [2].

In our studies, all sorbents, except for AF5, possess various quaternary ammonium or amine functional groups. They belong to strongly basic or weakly and weakly/chelating types of ion exchangers (see Table 1). On the other hand, the studies were carried out at pH 6 in acidic solutions (the forms of vanadium depending on its concentration and pH are included in Figure 2 in [11] and Figure 6a in this paper). For the anion exchange resin in form of Cl^−^, ion exchange reactions can occur (see Equations (7)–(10)) [3,54]. Moreover, electrostatic interactions can proceed.

### 2.4. Desorption Studies

Desorption of vanadium from the loaded sorbent (sorbent regeneration) and its reuse are an important factor influencing the practical application of sorbent and the total cost of the adsorption process. Vanadium is usually desorbed by various eluting agents, usually NaOH and HCl at various concentrations; e.g., vanadium is eluted from the SBA resins by 5% NaOH+10% NaCl (HZrO@ strongly basic anion exchanger D201, *%D* = 91.2%) [12], 6 mol/L HCl (D296 resin, *%D* = 98.68%) [8], 2 mol/L NaOH (Amberlite IRA400, *%D* = 57–72%, 30 min) [6], 2 mol/L NaOH (717, *%D* = 81.7%, 5 min) [55], or WBA resins by 3 mol/L NaOH (201*7, *%D* > 95%) [56], 2% NaOH+10% NaCl (Dex-V, *%D* = 99.5%) [14], etc. In such solutions, the repulsion between the vanadium oxyanions and the sorbents as well as the electrostatic attractions ceases between the protonated functional groups and vanadium, resulting in easier vanadium desorption. However, in many cases, vanadium removal is not quantitative, and desorption yields depend on the eluting type, regeneration time, temperature, and the type of mechanism [2].

In vanadium desorptions from the S984, A830, A400TL, SR7, PSR2, PSR3, and AF5 sorbents, the 0.1, 0.25, 0.5, and 1 mol/L HCl and 0.1, 0.25, 0.5, and 1 mol/L NaOH solutions were applied as eluting agents under experimental conditions: *W* = 0.1 ± 0.0005 g, *V* = 20 mL, pH 6, 170 rpm, *A* = 8, *T* = 295 K, *t* = 4 h (Table 6).

As observed, the desorption yield is changeable and depends on the eluting agent’s concentration. The desorption from SR7, PSR2, PSR3, and AF5 using the HCl solution is not satisfactory, and usually the largest desorption yield was obtained using 0.5 or 1 mol/L HCl for SR7, PSR2, PSR3, and 0.1 mol/L HCl for AF5. In the case of vanadium desorption from S984, A830, and A400TL, the desorption yield using 1 mol/L HCl is above 77.5%. Improved desorption was obtained using NaOH solutions, mainly the 0.5 mol/L NaOH solution. For the WBA resin, the desorption yield by NaOH is normally higher (quantitative) compared to SBA (*%D* is in the range from 5.3 to 99.7%), which is consistent with the literature data [6,12,14,56].

### 2.5. Column Studies

**Figure 11:**
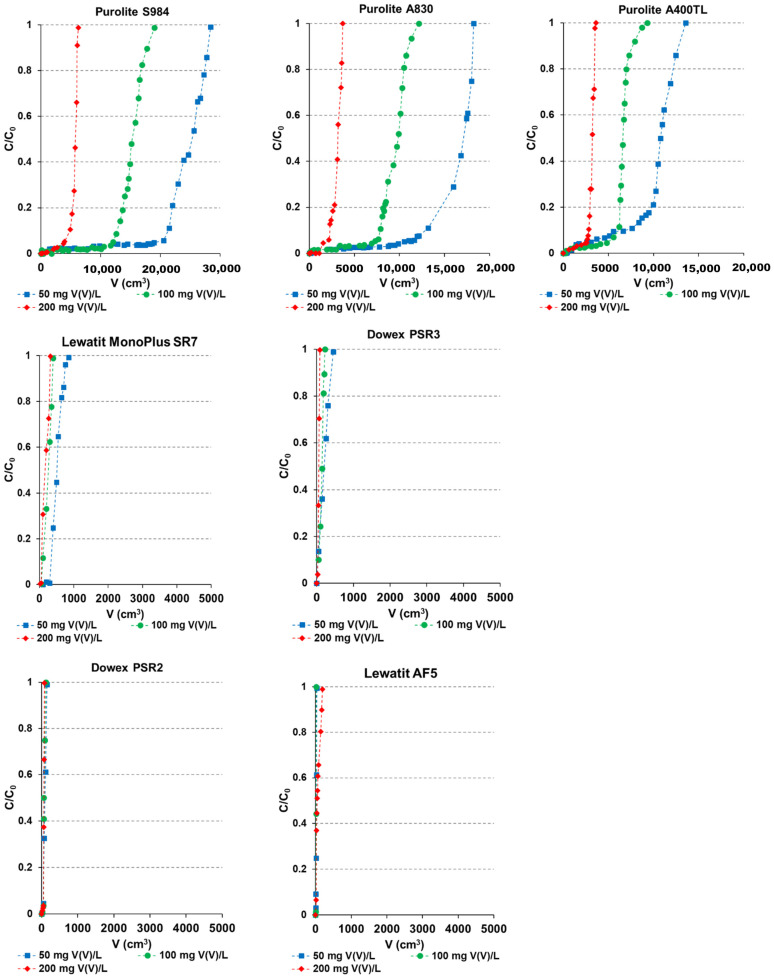
Breakthrough curves obtained during the adsorption of vanadium by the column studies.

The removal of contaminants in the continuous system is preferred in practical applications and is of great industrial importance. For this reason, the removal of vanadium from the solutions of its various initial concentrations (50, 100, and 200 mg/L) was conducted by the column method (pH 6, the solution flow rate through the bed was 0.4 mL/min). The eluate was collected in fractions of an appropriate volume until the initial concentration of V(V) ions was obtained. The column studies resulted in the determination of breakthrough curve (the ratio of V(V) concentrations in the aqueous solution (*C*) with an initial concentration of V(V) (*C*_0_) versus the eluate volume (*V*)) (Figure 11) and then the column parameters such as the weight distribution coefficient (*D_w_*), the bed distribution coefficient (*D_b_*), and the working ion exchange capacity (*C_w_*) (g/mL) were determined (see Equations (4)–(6) in Table 2 and results in Table 7).

As it was shown the breakthrough curves resembled the typical “S” shape in all cases. Moreover, the curve for the vanadium initial concentration 200 mg/L is steeper indicating that in this case the systems worked more efficiently. A reflection of this fact are the working ion exchange parameters values which increase with the increasing initial concentration and the highest values were obtained for the solutions of 200 mg V(V)/L. Based on the *C_w_* the efficiency series of the sorbents under discussion towards V(V) are:

*C*_0_ = 50 mg/L: S984 > A400TL > A830 > SR7 > PSR3 > PSR2 > AF5;
*C*_0_ = 100 mg/L: S984 > A830 > A400TL > SR7 > PSR3 > PSR2 > AF5;
*C*_0_ = 200 mg/L: S984 > A830 >A400TL > SR7 > PSR3 > PSR2 > AF5.

In all presented cases, the series are similar, indicating that for the AF5 sorbent being at the end of the series, column breakthrough occurs immediately after the adsorption process begins; therefore, the volume of the eluate collected to the breakthrough point is equal to 0, and the working ion exchange capacity is also zero. The weight and bed volume coefficients decrease with an increase in initial vanadium concentrations.

As it was found, S984, A830, and A400TL are the most efficient in vanadium removal by the column studies, which is consistent with the results obtained during equilibrium studies by means of the static method.

### 2.6. Removal of Vanadium from Real Wastewaters

The vanadium catalysts (4–9 wt% V_2_O_5_ together with the alkali metal sulfate promoters: potassium sulfate or cesium sulfate on the silica carrier material) are used for sulfuric acid production. The catalyst’s life cycle is limited from seconds even up to 10 years of work and then such a catalyst becomes a spent one (containing VOSO_4_, (VO_2_)_2_SO_4_ and V_2_O_5_) and can be either stored or processed to recover valuable metals [57,58]. The storage of spent catalysts requires appropriate conditions to protect them against weather conditions (the presence of sulfate, free sulphite, and water can result in an acidic leachate and environmental contamination) [57]. Therefore, the storage of spent catalysts is not environmentally acceptable, and their treatment by a simple process is preferable [59].

To study the sorbent’s applicability for vanadium removal from real wastewater, the vanadium spent catalyst for sulfuric acid production was leached using 5% m/m HCl (R1), 5% m/m H_2_SO_4_ (R2), and 15% m/m NaOH (R3), 15% m/m KOH (R4) (*T* = ambient, *t* = 6 h, S:L = 1.5). The characteristics of the real wastewater were previously described in [11]. After leaching, the content of V(V) was close to 5000 mg/L whereas that of Fe(III) was 50x higher in solutions obtained by digestion with the acid (1000 mg/L) than for the alkali ones (≈15–20 mg/L). After leaching, potassium, sodium, sulphur, chloride, silica, and copper(II) were also present in the solution.

It was found that the removal of V(V) and Fe(III) was the most effective by SR7, A400TL, PSR3, and PSR2 using 15% m/m KOH (R4) for spent catalyst digestion, whereas for WBA, resins (S984 and A830) V(V) and Fe(III) were the most efficiently removed from the R2 (5% m/m H_2_SO_4_) and R4 (15% m/m KOH) solutions, respectively. Lewatit AF5 did not reveal the applicability for V(V) removal, whereas the *%R* for Fe(III) was below 50.4% using alkali digestion agents. The largest removal of vanadium was obtained using the S984 ion exchanger (*%R* = 98%, R2), A830 (80.6%, R2), and A400TL (67.8%, R4). After pH adjustments to 5, in most cases, V(V) and Fe(III) removal efficiencies increased, and the precipitation of V(V) ions Fe(III) was observed in alkaline solutions.

## 3. Materials and Methods

### 3.1. Reagents and Instruments

All chemical compounds (sodium metavanadate, NaVO_3_, acids: HNO_3_, HCl, H_2_SO_4_; alkalis: NaOH, KOH) were of analytical grade and were supplied by Merc KGaA (Darmstadt, Germany) or Avantor Performance Materials (Gliwice, Poland).

The weakly basic (Purolite A830), weakly basic/chelating (Purolite S984), and strongly basic (Lewatit MonoPlus SR7, Purolite A400TL, Dowex PSR2, Dowex PSR3) ion exchangers and microporous Lewatit AF5 sorbents without the functional groups were purchased from Purolite International Co, Dow Chemical Company (Philadelphia, PA, USA) or Lanxess (Cologne, Germany). The sorbents were pre-treated before use and converted to the Cl^-^ form where necessary. The specifications of the sorbents under discussion are presented in Table 1.

The Fourier transform infrared spectra of the frequency range from 650 to 4000 cm^−1^ were obtained using the Attenuated Total Reflectance Fourier Transform Infrared Spectroscopy (ATR-FTIR) analysis by means of the Cary 630 instrument (Agilent Technologies, Santa Clara, CA, USA) and the Agilent MicroLab PC software (version: B.04) using the diamond attachment at a spectral resolution 4 cm^−1^ and the measurement time 30 s. The band intensities were expressed in transmittance (*%T*).

The SEM images of the sorbents were obtained using the Quanta^TM^ 3D FEG microscope with the EDS (Energy Dispersive Spectroscopy)/EBSD (Electron Backscatter Diffraction) system (Hillsboro, OR, USA).

The specific surface area (*S_BET_*), the total pore volumes (*P_v_*) and the average pore size (*P_s_*) were obtained by the Brunauer–Emmett–Teller method and by means of the ASAP 2405 analyzer (Micromeritics Instrument Corporation, Norcross, GA, USA).

All adsorption tests by the static method were carried out using the laboratory Elpin+ shaker type 358A (Lubawa, Poland).

After the adsorption process, V(V) concentrations in the solution were determined at a wavelength of 318.5 nm by applying the four-step time-temperature program (slit width 0.2 nm, lamp current 10 mA) by means of the Graphite Furnace Atomic Absorption Spectrometry (GF-AAS) and Varian AA240Z (Melbourne, Australia) spectrometer equipped with the graphite furnace tube GTA120.

### 3.2. Batch and Column Studies—Experimental Conditions

The vanadium(V) adsorption was conducted by means of static (batch) and dynamic (column) methods.

**Figure 12:**
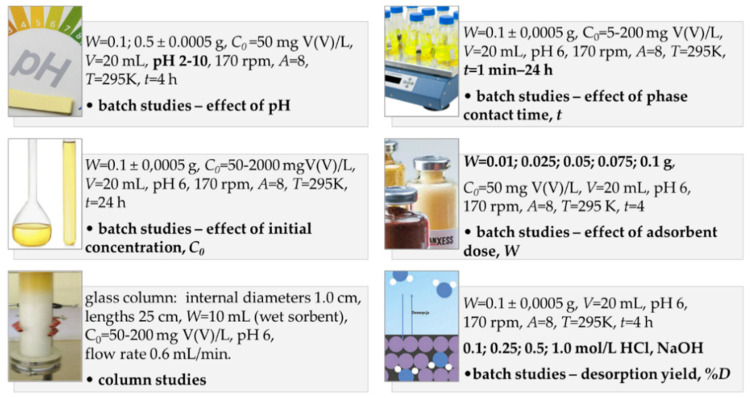
Experimental parameters applied in the vanadium(V) adsorption.

In the batch method, in the conical flasks with a 100 mL volume closed with a silicone stopper, 20 mL of V(V) solution of the initial concentrations at 50 mg/L (effect of pH, adsorbent dose), 5–200 mg/L (kinetic studies), 5–2000 mg/L (equilibrium studies), and 0.01–0.1 g (effect of adsorbent dose) (0.1, 0.5 g (effect of pH), or 0.1 g (others)) of the sorbent was placed. Then, Erlenmeyer flasks were inserted in a mechanical shaker operation at the vibration amplitude of 8 units and 170 cycles per minute at 295K and mixed from 1 min to 24 h (kinetic studies) for 4h (effect of pH, adsorbent dose, desorption) or 24 h (equilibrium studies). The details of experimental conditions are listed in Figure 12.

Then, the sorbent was separated from the solution, and the concentration of V(V) was measured using the GF-AAS method. Next the percentage removal (*%R*) (Equation (1)), the amount of V(V) adsorbed after time *t* (*q_t_*) (Equation (2)), and sorption capacity (*q_e_*) (Equation (3)) were calculated (Table 2).

Dynamic adsorption experiments were conducted in glass columns of the internal diameters equal to 1.0 cm and 25 cm in length. The columns were packed with the wet sorbent of 10 mL volume and then loaded with V(V) solutions of the initial concentrations 50, 100, and 200 mg/L and pH 6 at a flow rate 0.4 mL/min. The samples of proper volume were collected to determine the V(V) concentration and to obtain breakthrough curves. Then, the column parameters such as the weight (*D_w_*) (Equation (4)) and bed (*D_b_*) (Equation (5)) distribution coefficients as well as the working ion exchange capacity (*C_w_*) (Equation (6)) were calculated (Table 2).

The V(V) adsorption results were analyzed using kinetic models: the pseudo-first order kinetic equation (PFO), the pseudo–second order kinetic equation (PSO), the intraparticle diffusion (IPD) (Equations (11)–(15)) (kinetic studies results), or the isotherm ones—Langmuir, Freundlich, Temkin, and Dubinin–Raduskievich (Equations (16)–(25)) (equilibrium studies results).

The error functions such as the chi-square test (χ^2^) and the sum of squares of the errors (*SSE*) were applied for kinetic data analyses according to the equations presented in [60]:

(26) $$\chi^{2}=\overset{n}{\underset{i=1}{\sum }}\frac{{\left(q_{e\;exp}-q_{e\;cal}\right)}^{2}}{q_{e\;exp}}$$

(27) $$SSE=\overset{n}{\underset{i=1}{\sum }}{\left(q_{e\;exp}-q_{e\;cal}\right)}^{2}$$

where *q_e,exp_* (mg/g)—the experimental values of amount of V(V) adsorbed at equilibrium; *q_e,cal_* (mg/g)—the calculated amount of V(V) adsorbed.

## 4. Conclusions

In this study, the process of V(V) ion removal on the weakly basic (Purolite A830), weakly basic/chelating (Purolite S984), and strongly basic (Lewatit MonoPlus SR7, Purolite A400TL, Dowex PSR2, Dowex PSR3) ion exchange resins and the microporous Lewatit AF5 sorbent without functional groups using the static and column methods was investigated. The effects of pH, adsorbent dose, phase contact time, initial V(V) ions concentration on the V(V) adsorption efficiency, and the type and concentration of eluting agents on the desorption yield were discussed. In addition, the efficiency of V(V) and Fe(III) ions removal from real wastewater as a result of leaching of spent vanadium catalysts used in the production of sulfuric acid is presented. It was shown that the above mentioned parameters affect both the adsorption and desorption of V(V) ions on/from the tested sorbents significantly. The optimum pH and the sorbent’s dose were 6 and 0.1 g, respectively. Experimental adsorption data are well described by the PSO model and the Langmuir, Freundlich, or Temkin isotherm models. The obtained maximum experimental capacities (221.4 mg/L for S984, 202 mg/L for A830, and 200.4 mg/L for A400TL), working ion exchange capacities (25.6 g/mL for S984, 17.7 g/mL for A830, 12 g/mL for A400TL, *C*_0_ = 200 mg/L), the desorption efficiency (close to 100% using NaOH), and real wastewaters treatment for S984 (*%R* = 98%, R2), A830 (80.6%, R2), and A400TL (67.8%, R4) show that these ion exchangers can be promising in practical applications for vanadium(V) removal and vanadium containing wastewater treatment on a larger scale.

## Figures and Tables

**Table 1:** Physicochemical properties of SR7, A400TL, PSR2, PSR3, A830, S984, and AF5 sorbents.

Properties	SR7	A400TL	PSR2	PSR3	A830	S984	AF5	
Manufacturer	Lanxess	Lanxess	Dow	Dow	Purolite	Purolite	Lanxess	
Type	SBA	SBA	SBA	SBA	WBA	WBA/chelating	sorbent without FG	
Matrix	DVB cross-linked polystyrene	DVB cross-linked polystyrene	DVB cross-linked polystyrene	DVB cross-linked polystyrene	DVB cross-linked polyacrylic	DVB cross-linked polyacrylic	carbonaceous	
Structure	macroporous	microporous	microporous	macroporous	macroporous	macroporous	microporous	
Functional Groups (FG)	quaternary ammonium, type 3	quaternary ammonium, type 1	quaternary ammonium, tri-n-butyl amine		complex amine	polyamine *	without	
Size (mm)	0.57–0.67	0.425–0.85	0.3–1.2	0.3–1.2	0.3–1.2	-	0.4–0.8	
Max temp. (°C)	80	100 (Cl^−^)60 (OH^−^)	60	60	100 (Cl^−^)40 (OH^−^)	100 (Cl^−^)	300	
pH range	0–14	0–14	0–14	0–14	0–9	0–10	0–14	
Total capacity (eq/L)	0.6	1.3	0.65	0.6	2.75	2.7	-	
Water retention (%)	59–64	48–54	40–48	50–65	47–53	45–55	48–60	
Appearance	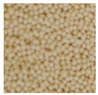	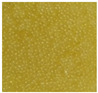	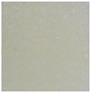	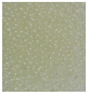	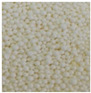	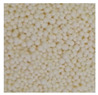	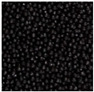	
%N	2.58	4.54	2.49	2.25	16.35	18.13	0.02	[11]
%C	70.79	59.64	71.38	73.89	46.00	46.95	93.81	
%H	6.49	9.76	8.02	10.89	8.39	7.62	1.58	
%S	0.26	0.24	1.28	0.06	0.00	0.87	0.91	
*S_BET_* (m^2^/g)	19.69	4.20	6.32	6.36	9.66	3.80	988.8	
*P_s_* (nm)	44.61	2.35	10.08	3.65	48.83	89.36	2.31	
*P_v_* (cm^3^/g)	0.220	0.002	0.016	0.006	0.012	0.085	0.572	
pH_PZC_	1.48	1.00	1.00	1.03	6.05	9.01	7.09	[15]

WBA—the weakly basic anion exchanger; SBA—the strongly basic anion exchanger; DVB—divinylbenzene; * mixed primary, secondary, and tertiary amines; *S_BET_*—the BET surface area; *P_s_*—the average pore size; *P_v_*—the total pore volume.

**Table 2:** Adsorption efficiency determination by static and dynamic studies.

Parameters	Equation	No.
**Static method**		
percentage removal *(%R*)	$\%R=\frac{C_{0}-C_{t}}{C_{0}}\times 100\%$	(1)
sorption capacity (*q_e_*) (mg/g)	$q_{e}=\frac{(C_{o\;}-C_{e})\times V}{m}$	(2)
amount of V(V) adsorbed after time *t* (*q_t_*) (mg/g)	$q_{t}=\frac{(C_{0}-C_{t})\times V}{m}$	(3)
**Dynamic method**		
weight distribution coefficient (*D_w_*)	$D_{w}=\frac{U^{”}-U_{0}-V_{f}}{m_{j}}$	(4)
bed distribution coefficient (*D_b_*)	$D_{b}=D_{w}\times d_{z}$	(5)
working ion exchange capacity (*C_w_*) (g/mL)	$C_{w}=\frac{U_{p}\times C_{0}}{V_{j}}$	(6)

*C*_0_, *C_e_*, *C_t_*—the initial, equilibrium, and after time *t* concentration of V(V) in the water phase (mg/L); *V*—the volume of solution; *m*—the mass of dry sorbent; *U”, U*_0_—the eluate volume for *C/C*_0_ = 0.5 (mL) and the dead column volume (2 mL); *V_f_*—the free volume of the sorbent bed (4 mL); *m_j_*—the mass of dry sorbent in the column (g); *d_z_*—the sorbent density (g/mL); *U_p_*—the volume of eluate to the break through the column (L); *C*_0_ in Equation (6)—the initial V(V) concentration in the solution (g/L); *V_j_*—the volume of the sorbent in the column.

**Table 3:** Comparison of kinetic parameters obtained during the V(V) adsorption.

Parameters		A830			S984			A400TL			SR7	PSR2	PSR3
*C*_0_ *(mg/L)*		50	100	200	50	100	200	50	100	200	200	200	200
*q_e, exp_*		8.77	18.09	40.38	8.30	16.95	39.10	8.82	19.34	41.54	40.52	38.88	23.92
**PFO-L**	*q_e, cal_*	4.88	10.35	32.04	3.94	8.46	28.27	0.62	3.44	33.91	10.30	14.23	17.04
	*k*_1_	0.022	0.012	0.007	0.013	0.014	0.013	0.019	0.005	0.021	0.025	0.007	0.004
	*R* ^2^	0.957	0.906	0.985	0.906	0.896	0.959	0.710	0.439	0.954	0.959	0.904	0.909
**PFO-NL**	*q_e, cal_*	7.56	16.32	23.83	7.40	16.54	35.18	8.45	17.90	44.18	37.95	30.03	12.85
	*k*_1_	0.137	0.066	0.088	0.104	0.058	0.043	0.772	0.384	0.015	0.384	0.561	0.147
	*R* ^2^	0.865	0.950	0.645	0.886	0.991	0.962	0.798	0.938	0.960	0.853	0.429	0.610
	*R* ^2^ *_adj_*	0.834	0.939	0.566	0.861	0.989	0.953	0.753	0.924	0.951	0.821	0.302	0.524
	*MPSD*	0.379	0.638	1.359	0.645	0.062	0.411	0.067	0.061	0.398	0.185	0.455	0.695
	*χ* ^2^	1.79	2.93	24.79	2.06	0.39	4.58	13.40	29.47	19.89	4.22	12.43	11.14
	*SSE*	11.36	21.23	690.39	8.45	3.94	92.82	92.11	392.62	237.27	106.95	367.31	212.68
**PSO-L**	*q_e, cal_*	8.83	18.26	41.22	8.35	17.11	39.74	8.82	19.35	43.10	40.59	39.02	24.25
	*k*_2_	0.017	0.004	0.001	0.015	0.006	0.001	0.178	0.010	0.001	0.014	0.003	0.001
	*R* ^2^	1.000	1.000	0.996	1.000	1.000	1.000	1.000	1.000	0.996	1.000	1.000	0.991
	*h*	1.32	1.49	1.04	1.07	1.62	2.10	13.87	3.76	1.16	22.98	4.22	0.49
**PSO-NL**	*q_e, cal_*	8.33	17.77	26.63	7.98	18.53	39.51	8.83	18.88	54.40	39.79	32.74	14.82
	*k*_2_	0.020	0.004	0.004	0.017	0.003	0.001	0.138	0.028	0.000	0.015	0.022	0.010
	*MPSD*	0.1245	0.4581	0.9113	0.416	0.139	0.225	0.009	0.009	0.603	0.058	0.226	0.410
	*R* ^2^	0.963	0.980	0.768	0.947	0.972	0.988	0.973	0.984	0.909	0.960	0.727	0.765
	*R* ^2^ *_adj_*	0.952	0.975	0.702	0.936	0.966	0.985	0.967	0.981	0.888	0.951	0.666	0.712
	*χ* ^2^	0.55	1.75	16.73	3.78	8.33	12.15	0.06	0.15	12.86	1.25	5.91	6.78
	*SSE*	3.31	8.72	471.92	11.37	74.72	184.29	0.45	2.53	387.61	28.22	167.68	131.14
**IPD**	*q_e, cal_*	34.22	73.36	71.14	32.79	84.76	88.06	58.62	147.0	135.4	50.25	49.01	32.45
	*k_i_*	0.87	1.91	1.72	0.83	2.24	2.01	1.44	3.76	3.48	0.39	0.54	0.69
	*R* ^2^	0.943	0.967	0.995	0.979	0.908	0.913	0.960	0.950	0.901	0.860	0.859	0.948
	*R* ^2^ *_adj_*	0.914	0.950	0.993	0.968	0.862	0.738	0.881	0.900	0.703	0.581	0.577	0.922

*q_e, exp_* (mg/g), *q_e, cal_* (mg/g), *k*_1_ (1/min), k_2_ (g/mg min), *k_i_* (mg/g min^0.5^).

**Table 4:** Kinetic and equilibrium studies for vanadium on ion exchangers and biochar—comparison.

Adsorbent	Kinetic Studies	Isotherm Studies	Ref.
* linear, ** non-linear regression			
Amberlite^®^ IRA-400	*W* = 10 g; *V* = 150 mL; 150 rpm; *t* = 1–30 min; *T* = 20 °C Post-closure effluent: *C*_0_ = 5.3 mg/L; pH 11.5 Op. BRDA leachate: *C*_0_ = 4.7 mg/L; pH 13.3 * **PFO** Post-closure effluent: *k*_1_ = 1.181 1/min; *R*^2^ = 0.977 Op. BRDA leachate: *k*_1_ = 0.545 1/min; *R*^2^ = 0.985	*V* = 20 mL; *C*_0_ = 1–50 g/L; 150 rpm; *t* =30 min; *T* = 20 °C Langmuir (L), **Freundlich (F)** Post-closure effluent: pH 11.5 L: *q_max_* = 1.135 mg/g; *b* = 3.218 L/mg; *R*^2^ = 0.859, F: *k_F_* = 0.547; *n* = 2.383; *R*^2^ = 0.999, Op. BRDA leachate: pH 13.3 L: *q_max_* = 9.759 mg/g; *b* = 0.060 L/mg; *R*^2^ = 0.919, F: *k_F_* = 0.285; *n* = 1.050; *R*^2^ = 0.993	[36]
Lewatit MonoPlus TP220; Dowex M4195	*W* = 0.1 ± 0,0005 g; *C*_0_ = 50; 100; 200 mg V(V)/L; *V* = 20 mL; pH 4; 170 rpm, *A* = 8; *T* = 295 K, *t* = 1 min–24 h *, ** PFO, **PSO**, IPD *C*_0_ = 50 mg/L, * PSO: *q_e cal_* = 7.68 mg/g; *k*_2_ =0.018 g/mg min; *R*^2^ = 0.998, TP220 *C*_0_ = 50 mg/L, * PSO: *q_e cal_*=9.54 mg/g; *k*_2_ = 0.021 g/mg min; *R*^2^ = 0.999, M4195	*W* = 0.1 ± 0.0005 g; *C*_0_ = 50–2000 mg V(V)/L; *V* = 20 mL; pH 4; 170 rpm, *A* = 8; *T* = 295 K; *t*=24 h *, ** **Langmuir (L)**, Freundlich (F), Temkin (T), Dubinin-Radushkevich (D-R) * L: *q_max_* = 247.8 mg/g; k_L_ = 0.014 L/mg; *R*^2^ = 0.963, TP220; * L: *q_max_* = 208.8 mg/g; k_L_ = 0.037 L/mg; *R*^2^ = 0.998, M4195 **F and L, TP220; ** L and T, M4195	[11]
Amberlite IRA743; Lewatit MK51; Purolite S110; Purolite S108	1 g/L; *C*_0_ = 10–100 mg V(V)/L; pH 5; 180 rpm, *A* = 7; *T* = 293 K, *t* = 1–240 min ** PFO, **PSO**, IPD, Boyd *C*_0_ = 50 mg/L: ** PSO: *q_e cal_* = 31.28 mg/g; *k*_2_ = 0.0017 g/mg min; *R*^2^ = 0.978, IRA473 ** PSO: *q_e cal_* = 32.35 mg/g; *k*_2_ = 0.0015 g/mg min; *R*^2^ = 0.992, MK51 ** PSO: *q_e cal_* = 39.18 mg/g; *k*_2_ = 0.0014 g/mg min; *R*^2^ = 0.988, S110 ** PSO: *q_e cal_* = 41.06 mg/g; *k*_2_ = 0.0021 g/mg min; *R*^2^ = 0.994, S108 ** PFO *R*^2^ = 0.882–0.999; IPD *R*^2^ = 0.933–0.993, Boyd 0.947–0.998 for all resin and concentration	1 g/L; *C*_0_ = 10–150 mg V(V)/L; pH 5; *T*=293, 313, 333 K, *t* = 240 min ** **Langmuir (L), Freundlich (F),** Temkin (T) at 333K: ** L: *q_max_* = 68.27 mg/g; *k_L_* = 0.122 L/mg; *R*^2^ = 0.940, IRA473; ** L: *q_max_* = 72.51 mg/g; *k_L_* = 0.150 L/mg; *R*^2^ = 0.975, MK51; ** L: *q_max_* = 66.42 mg/g; *k_L_* = 0.277 L/mg; *R*^2^ = 0.989, S110; ** L: *q_max_* = 77.03 mg/g; *k_L_* = 0.416 L/mg; *R*^2^ = 0.992, S108; at 293, 313, 333 K: L: *R*^2^ = 0.992–0.997; F: *R*^2^ = 0.958–0.998; T: *R*^2^ = 0.802–0.996	[17]
Tulsion A-62	*W* = 0.1 g; *V* = 40 mL; 160 rpm, *t* = 10–200 min *%R* = 70% (15 min), *%R* = 97.5% (180 min) no kinetic model was used	* **Langmuir (L), Freundlich (F)** L: *q_max_* = 36.9 mg/g; *k_L_* = 0.585 1/mol; *R*^2^ = 0.964; F: *k_F_* = 15.33 mg/g; *n* = 3.2 L/g; *R*^2^ = 0.980	[46]
biochars BC2	*W*=0.5 g; *C*_0_ =10–50 mg V(V)/L; *V*=20 mL; pH 3; *t*=5–360 min * PFO, **PSO**, IPD *C*_0_ = 50 mg/L: *PSO: *q_e cal _*= 0.83 mg/g; *k*_2_ =0.07 g/mg min; *R*^2^ = 0.999 *C*_0_ = 10–50 mg/L: *PFO *R*^2^ = 0.912–0.990; IPD *R*^2^=0.816–0.997	*W*=0.5 g; *C*_0_ = 5–150 mg V(V)/L; *V* = 20 mL; pH 3; *t* = 300 min, *T* = 298K * Langmuir (L), **Freundlich (F)**, Temkin (T), Dubinin-Radushkevich (D-R) * L: *q_max_* = 0.52 mg/g; *k_L_* = 0.05 L/mg; *R*^2^ = 0.974 * F: *k_F_* = 0.06 mg/g; *n* = 1.96 L/g; *R*^2^ = 0.998 * T: *k_T_* = 0.82 L/g; *b_T_* = 21.62 kJ/mol; *R*^2^ = 0.952 * D-R: *q_m_* = 0.31 × 10^−4^ mol/g; *K_DR_* = 4.7 × 10^−3^ mol^2^/kJ^2^; *R*^2^ = 0.993	[4]
Purolite A830, Purolite S984, Purolite A400TL, Lewatit MonoPlus SR7, Dowex PSR2, Dowex PSR3, Lewatit AF5	*W* = 0.1 ± 0,0005 g; *C*_0_ = 50; 100; 200 mg V(V)/L; *V* = 20 mL; pH 6; 170 rpm, *A* = 8; *T* = 295 K, *t* = 1 min–24 h *, ** PFO, **PSO**, IPD *C*_0_ = 50 mg/L: * PSO: *q_e cal_* = 8.83 mg/g; *k*_2_ = 0.017 g/mg min; *R*^2^ = 1.000, A830; * PSO: *q_e cal_* = 8.35 mg/g; *k*_2_ = 0.015 g/mg min; *R*^2^ = 1.000, S984; * PSO: *q_e cal_* = 8.82 mg/g; *k*_2_ = 0.178 g/mg min; *R*^2^ = 1.000, A400TL; * PSO: *q_e cal_* = 8.79 mg/g; *k*_2_ = 0.055 g/mg min; *R*^2^ = 1.000, SR7; * PSO: *q_e cal_* = 8.02 mg/g; *k*_2_ = 0.005 g/mg min; *R*^2^ = 0.997, PSR2; * PSO: *q_e cal_* = 8.77 mg/g; *k*_2_ = 0.039 g/mg min; *R*^2^ = 1.000, PSR3; AF5-no kinetic model was used	*W* = 0.1 ± 0,0005 g; *C*_0_ = 50; 100; 200 mg V(V)/L; *V* = 20 mL; pH 6; 170 rpm, *A* = 8; *T* = 295 K, *t* = 24 h *, ** **Langmuir (L), Freundlich (F), Temkin (T)**, Dubinin-Radushkevich (D-R) * L: *q_max_* = 204.1 mg/g; k_L_ = 0.098 L/mg; *R*^2^ = 1.000, A830 * L: *q_max_* = 256.62 mg/g; k_L_ = 0.008 L/mg; *R*^2^ = 0.927, S984; * L: *q_max_* = 204.27 mg/g; k_L_ = 0.0049 L/mg; *R*^2^ = 0.999, A400TL; * L: *q_max_* = 39.49 mg/g; k_L_ = 0.045 L/mg; *R*^2^ = 0.959, SR7; * L: *q_max_* = 40.42 mg/g; k_L_ = 0.024 L/mg; *R*^2^ = 0.940, PSR2; * L: *q_max_* = 39.57 mg/g; k_L_ = 0.31 L/mg; *R*^2^ = 0.955, PSR3; * F: *k_F_* = 2.00 mg^1−1/n^ L^1/n^/g; *1/n* = 0.63; *R*^2^ = 0.972, AF5	This paper

Op. BRDA leachate—operating bauxite residue disposal areas leachate; **bold**—the best fit; BC2—wet distiller grains.

**Table 5:** Characterization of the isotherm models and the parameters obtained for the V(V) ions sorption on the S984, A830, A400TL, SR7, PSR2, PSR3, and AF5 sorbents.

Isotherm	Non-linear Forms		Equation		Linear Forms			Equation
Langmuir	$q_{e}=\frac{k_{L}Q_{0}C_{e}}{1+C_{e}k_{L}}$		(16)		$\frac{C_{e}}{q_{e}}\;=\;\frac{1}{Q_{0}k_{L}}+\frac{C_{e}}{Q_{0}}$			(17)
Freundlich	$q_{e}=k_{F}C_{e}^{1/n}$		(18)		$log\;q_{e}=log\;k_{F}+\frac{1}{n}log\;C_{e}$			(19)
Temkin	$q_{e}=\frac{RT}{b_{T}}lnAC_{e}$		(20)		$q_{e}=\left(\frac{RT}{b_{T}}\right)lnA+\left(\frac{RT}{b_{T}}\right)lnC_{e}$			(21)
Dubinin-Radushkevich	$q_{e}=q_{m}e^{k_{DR}\varepsilon^{2}}$		(22)		$lnq_{e}=lnq_{m}-k_{DR}\varepsilon^{2}$			(25)
	$\varepsilon =RTln\left[1+\frac{1}{C_{e}}\right]$		(23)					
	$E=\frac{1}{\sqrt{2k_{DR}}}$		(24)					
	**Isotherm Parameters**							
Model	Parameters	S984	A830	A400TL	SR7	PSR2	PSR3	AF5
	*Q_e, exp_.* (mg/g)	221.4	202.00	200.4	30.80	29.28	29.12	27.76
Langmuir	*Q_0_* (mg/g)	256.62	204.10	204.27	39.49	40.42	39.57	37.98
	*k_L_* (L/mg)	0.008	0.098	0.049	0.045	0.024	0.031	0.035
	*R^2^*	0.927	1.000	0.999	0.959	0.940	0.955	0.772
Freundlich	*k_F_* (mg^1−1/n^ L^1/n^/g)	5.58	24.19	19.87	1.84	1.13	1.37	2.00
	*1/n*	0.613	0.377	0.395	0.722	0.766	0.745	0.630
	*R^2^*	0.691	0.820	0.769	0.963	0.942	0.962	0.972
Temkin	*b_T_* (J g/mol mg)	52.40	87.12	82.78	386.72	394.73	397.73	537.26
	*A* (L/mg)	0.170	2.730	1.466	1.198	0.668	0.847	1.806
	*R^2^*	0.904	0.947	0.960	0.897	0.954	0.924	0.793
Dubinin–Raduskievich	*q_m_* (mg/g)	159.50	118.45	136.78	15.99	14.39	13.24	10.70
	*k_DR_* (mol^2^J^2^)	6.1 × 10^−5^	3.76 × 10^−7^	1.57 × 10^−6^	4.40 × 10^−7^	11.3 × 10^−7^	5.16 × 10^−7^	1.31 × 10^−7^
	*E* (kJ/mol)	0.091	1.154	0.565	1.068	0.665	0.985	1.957
	*R^2^*	0.921	0.694	0.774	0.747	0.736	0.596	0.513

*q*_e_ (mg/g)—the amount of V(V) ions sorbed per unit mass of sorbent; *C*_e_ (mg/L)—the equilibrium concentration of solution; *Q_0_* (mg/g)—the monolayer adsorption capacity; *k_L_* (L/mg)—the Langmuir constant (related to the free energy of adsorption); *k*_F_ (mg^1−1/n^ L^1/n^/g) and 1/*n*—the Freundlich constants connected with the adsorption capacity of the adsorbent and the surface heterogeneity; R (8.314 J/mol K)—the gas constant; *T* (K)—the temperature; *A* (L/g) and *b_T_* (J/mol)—the Temkin constants; *q_m_* (mg/g)—the maximum adsorption capacity; *k_DR_* (mol^2^ J^2^)—the constant related to the adsorption energy; *ε* (J/mol)—the adsorption potential; *E* (J/mol)—the mean free energy for the V(V) ions removal from adsorption site to the infinity.

**Table 6:** Vanadium desorption yield from S984, A830, A400TL, SR7, PSR2, PSR3, AF5.

	S984	A830	A400TL	SR7	PSR2	PSR3	AF5
0.1 mol/L HCl	57.6	27.1	5.9	6.5	5.0	4.3	15.5
0.25 mol/L HCl	85.8	42.7	15.0	6.1	5.7	2.4	11.0
0.5 mol/L HCl	≈ 100%	59.1	26.0	7.4	8.8	6.1	11.2
1 mol/L HCl		76.6	77.5	14.4	5.4	7.6	11.5
0.1 mol/L NaOH		≈ 100%	45.5	66.6	6.4	58.8	11.5
0.25 mol/L NaOH			70.6	82.6	6.2	58.8	19.5
0.5 mol/L NaOH			99.9	84.2	7.0	60.4	26.8
1 mol/L NaOH			99.7	78.2	5.3	59.4	14.8

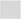
 The largest desorption yield.

**Table 7:** Column parameters obtained for the vanadium adsorption from the solutions of the initial concentrations 50, 100, and 200 mg/L.

Sorbent	C_w_ (g/mL)	*D_w_*	*D_v_*
A830	3.5	4914.9	1710.4
	9.5	2831.6	985.4
	17.7	2629.0	914.9
A400TL	4.5	3172.7	1074.9
	8.0	1946.3	659.4
	12.0	940.7	318.7
S984	7.0	6755.4	2529.9
	23.0	4082.5	1528.9
	25.6	1543.9	578.2
SR7	1.0	190.7	50.7
	1.0	95.1	25.3
	1.0	59.8	15.9
PSR3	0.3	63.6	19.9
	0.5	44.7	14.0
	0.5	16.9	5.3
PSR2	0.1	22.0	8.5
	0.3	15.2	5.9
	0.4	15.0	5.8
AF5	0	3.6	2.1
	0	1.6	1.0
	0	7.2	4.2

## Data Availability

The data presented in this study are available in this article.

## Sample Availability

Samples of the compounds are not available from the authors.
